# Antifungal Effect of Antimicrobial Photodynamic Therapy Mediated by Haematoporphyrin Monomethyl Ether and Aloe Emodin on *Malassezia furfur*

**DOI:** 10.3389/fmicb.2021.749106

**Published:** 2021-11-16

**Authors:** Zixin Cui, Miaomiao Zhang, Songmei Geng, Xinwu Niu, Xiaopeng Wang, Yanyan Zhu, Feng Ye, Chengcheng Liu

**Affiliations:** ^1^Department of Infection, The First Affiliated Hospital of Xi’an Jiaotong University, Xi’an, China; ^2^Department of Pathogenic Microbiology and Immunology, School of Basic Medical Sciences, Xi’an Jiaotong University Health Science Center, Xi’an, China; ^3^Department of Dermatology, The Second Affiliated Hospital of Xi’an Jiaotong University, Xi’an, China

**Keywords:** *Malassezia furfur*, photodynamic antimicrobial effect, haematoporphyrin monomethyl ether, aloe emodin, protease and lipase

## Abstract

Infectious dermatological diseases caused by *Malassezia furfur* are often chronic, recurrent, and recalcitrant. Current therapeutic options are usually tedious, repetitive, and associated with adverse effects. Alternatives that broaden the treatment options and reduce side effects for patients are needed. Antimicrobial photodynamic therapy (aPDT) is an emerging approach that is quite suitable for superficial infections. The aim of this study is to investigate the antimicrobial efficacy and effect of aPDT mediated by haematoporphyrin monomethyl ether (HMME) and aloe emodin (AE) on clinical isolates of *M. furfur in vitro*. The photodynamic antimicrobial efficacy of HMME and AE against *M. furfur* was assessed by colony forming unit (CFU) assay. The uptake of HMME and AE by *M. furfur* cells was investigated by fluorescence microscopy. Reactive oxygen species (ROS) probe and flow cytometry were employed to evaluate the intracellular ROS level. The effect of HMME and AE-mediated aPDT on secreted protease and lipase activity of *M. furfur* was also investigated. The results showed that HMME and AE in the presence of light effectively inactivated *M. furfur* cells in a photosensitizer (PS) concentration and light energy dose-dependent manner. AE exhibited higher antimicrobial efficacy against *M. furfur* than HMME under the same irradiation condition. HMME and AE-mediated aPDT disturbed the fungal cell envelop, significantly increased the intracellular ROS level, and effectively inhibited the activity of secreted protease and lipase of *M. furfur* cells. The results suggest that HMME and AE have potential to serve as PSs in the photodynamic treatment of dermatological diseases caused by *M. furfur*, but further *ex vivo* or *in vivo* experiments are needed to verify that they can meet the requirements for clinical practice.

## Introduction

*Malassezia furfur* is a lipid-dependent yeast commonly found on the skin of animal and human as normal microbiota. This fungus normally accounts for more than 80% of the total fungal population on human skin and can be frequently isolated from healthy and diseased hosts (Gao et al., 2010). In most cases, *M. furfur* is associated with the maintenance of skin health (Ashbee and Evans, 2002; Prohic et al., 2016). But under appropriate conditions, it invades the stratum corneum, interacts with host immune system, and causes various infectious dermatological diseases, including *Malassezia* folliculitis, pityriasis versicolor, and seborrheic dermatitis (Saunte et al., 2020). Although the pathogenic mechanism of such diseases has not been identified, the current hypotheses suggest that these diseases might be induced directly by invasion of fungal filaments in skin tissue, or indirectly through immunological and metabolic mechanisms caused by this fungus (Spatz and Richard, 2020). Additionally, *M. furfur* is also associated with psoriasis, Parkinson’s disease, onychomycosis, and systemic fungal infections (Gaitanis et al., 2012; Torres et al., 2020).

At present, available therapeutic options for skin diseases caused by *M. furfur* are mainly depending on systemic and topical antifungal agents, such as azoles, polyenes, allylamines, and echinocandins (Rhimi et al., 2021). Although these antifungals are effective, they are still associated with several adverse effects, e.g., hepatotoxicity, gastrointestinal discomfort, high risk of drug interactions, burning, stinging, and redness. Besides, the treatment process is usually tedious and repetitive because *M. furfur*-caused skin diseases are chronic and recurrent (Takahashi et al., 2014). And a great number of patients who have comorbidities or contraindications to oral medications cannot use systemic antifungal drugs (Takahashi et al., 2014). Moreover, the irregular and continuous use of these antifungals can result in the emergence of drug-resistant strains (Cafarchia et al., 2015; Iatta et al., 2017). Thus, alternative approaches that broaden the treatment options and reduce side effects for patients are needed.

Antimicrobial photodynamic therapy (aPDT) has emerged as a promising method to kill various microorganisms and has been explored for the treatment of infectious skin diseases (Li et al., 2020). When a photosensitizer (PS) is applied to the infected site of skin followed by irradiation with specific wavelength of light, the PS is excited from the ground singlet state to triplet state that reacts with molecular oxygen around or inside the microbial cells to form reactive oxygen species (ROS), such as superoxide anions, hydroxyl radicals, hydrogen peroxide, and primarily singlet oxygen (Hamblin and Hasan, 2004; Jia et al., 2017b). These ROS can induce irreversible oxidative damage to many intracellular structures (cell wall, cytoplasm membrane, and organelles) or important biomolecules (cellular DNA, proteins, and membrane lipids) of microbial cells, which ultimately leads to their death (Calzavara-Pinton et al., 2012; Dai et al., 2012; Jia et al., 2017b).

With respect to conventional antifungal drugs, aPDT possesses following benefits: (1) all investigated PSs exhibit no significant mutagenic and genotoxic activity (Calzavara-Pinton et al., 2012); (2) microbial cells can be inactivated at the concentrations of PSs and energy doses of light much lower than that needed for a same effect on keratinocytes (Calzavara-Pinton et al., 2012); and (3) it is generally believed that microbial cells impossibly develop resistance to aPDT because the oxidative action of ROS is non-specific and oxidative damage occurs on many intracellular targets (Kashef and Hamblin, 2017). For these benefits, aPDT has been employed to treat skin diseases caused by many yeasts, such as sporotrichosis, chromoblastomycosis, tinea pedis, tinea cruris, and onychomycosis, etc. (Baltazar et al., 2016). Nevertheless, the treatment effect of aPDT on skin diseases caused by *M. furfur* has been seldom investigated *in vitro* and *in vivo*.

**FIGURE 1:**
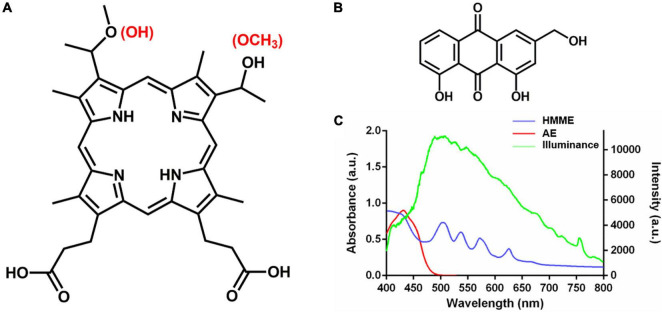
Chemical structure and absorption spectrum of HMME and AE. **(A)** Chemical structure of HMME; **(B)** chemical structure of AE; and **(C)** the absorption spectrum of HMME and AE and the spectrum of light source.

Haematoporphyrin monomethyl ether (HMME, Figures 1A,C) is a second-generation porphyrin monomer PS developed in China, which has been approved by the China Food and Drug Administration for photodynamic treatment of port wine stain (naevus flammeus; Tang et al., 2017). Aloe emodin (AE, Figures 1B,C) is a natural anthraquinone PS extracted from Chinese traditional herbs *Aloe vera*, *Polygonum multiflorum*, *Cassia occidentalis*, and *Rheum palmatum* L (Dong et al., 2020). We previously found that aPDT mediated by the two PSs from China could effectively inactivate *Candida albicans* and *Trichophyton rubrumin vitro* and *in vivo* (Liu et al., 2016; Ma et al., 2020, 2021; Pan et al., 2020). To evaluate the potential of these PSs for photodynamic treatment of skin diseases caused by *M. furfur*, clinical isolates were employed to investigate the photodynamic antimicrobial efficacy and effect of HMME and AE in this work.

## Materials and Methods

### Photosensitizers and Light Instruments

HMME and AE were purchased from China Shanghai Xianhui Pharmaceutical Co. and China Nanjing Jingzhu Biotechnology Co., respectively. To prepare PSs solutions, 6.13 mg of HMME and 2.70 mg of AE were measured and 50 μL of DMSO was added. The two PSs were completely dissolved in DMSO using micropipettor followed by sonication at 45°C for 10 s. Next, 950 μL of pre-heated (45°C) sterile PBS were added into DMSO in batches, and each diluted PSs solutions were sonicated at 45°C for three times (10 s each time) and stirred at 45°C until transparency. Subsequently, the PSs solutions were diluted to the desired concentrations with 45°C sterile PBS. After cooling to room temperature, the prepared PSs solutions were applied in photodynamic experiments immediately.

Light irradiations were conducted on a 50-W xenon lamp (Ceaulight CEL-HXF300, China). To avoid ultra-violet and near-infrared light, an optical filter was equipped on the xenon lamp for selecting of 400–780 nm white light, and the spectra of illumination was measured by a fiber optic spectrometer (Ocean Optics HR4000, United States) at the level of samples (Figure 1C). A power meter (Ceaulight NP-2000, China) was employed to adjust the light fluence rate at the level of samples to 40 mW cm^–2^.

### Clinical Isolates of *M. furfur* and Culture Conditions

Four clinical isolates of *M. furfur* used in this study were collected from anonymous patients in the Department of Dermatology, the Second Affiliated Hospital of Xi’an Jiaotong University, Xi’an, China. Clinical isolate 1 was obtained from a patient with *Malassezia* folliculitis. Clinical isolate 2 and isolate 3 were collected from the patients with pityriasis versicolor. Clinical isolate 4 was obtained from a patient with seborrheic dermatitis. All isolates were firstly identified by morphological observation of the fungal cells stained with lactophenol cotton blue by using an optical microscope, and characterized by developing cream-colored and pink umbonate round colonies on Sabouraud dextrose agar (SDA, Solarbio, China) supplemented with 2% olive oil and CHROMagar^TM^ *Candidia* agar (CHROMagar, France) after incubation, and further confirmed by biochemical methods (catalase test and Escalin decomposing test; Cafarchia et al., 2011; Chen and Xu, 2012).

The four clinical isolates were spread onto olive oil agar plate (containing 40 g/L glucose, 10 g/L peptone, 0.05 g/L chloramphenicol, 0.1 g/L yeast extract, 0.2% Tween 80, 4% olive oil, 2.5 g/L glycerin monostearate, and 18 g/L agar) and cultured at 37°C for 4 days. After incubation, fungal cells were collected by swabbing the surface of developed colonies with a sterile inoculation loop and transferred into 15 mL Falcon tube (Corning, United States) containing 10 mL of sterile PBS. Following three times wash with sterile PBS, fungal cells were diluted to a density of 1 × 10^7^ colony forming units (CFU)/mL with a hemocytometer (Marienfeld, Germany).

### Photodynamic Inactivation

Fungal cells of the four clinical isolates (1 × 10^7^ CFU/mL) were collected by centrifugation at 4,000 rpm for 10 min (Beckman, United States) and divided into two groups. The first group fungal cells were incubated with different concentrations (0, 0.5, 1, 5, and 10 μM) of HMME or AE in the dark for 30 min. Then, the incubated suspensions were transferred into the wells of a 6-well polystyrene microplate (Corning, United States) and irradiated with 400–780 nm white light for 20 min, corresponding to the total light energy dose of 96 J cm^–2^. The second group fungal cells were incubated with 10 μM of HMME or AE in the dark for 30 min and irradiated with 400–780 nm white light for 0, 0.5, 5, 10, and 20 min, corresponding to the total light energy doses of 0, 2.4, 24, 48, and 96 J cm^–2^, respectively. Following illuminations, cells were centrifuged, resuspended in sterile PBS, and 10-fold diluted serially with sterile PBS. Next, 20 μL of each dilution was evenly spread onto three olive oil agar plates, and the colonies after incubation at 37°C for at least 48 h were counted and used to calculate the fungal survival. Each experiment was performed independently for three times.

### Fluorescence Microscopy

Fungal cells of clinical isolate 1 and isolate 2 (1 × 10^7^ CFU/mL) were collected by centrifugation, incubated with 10 μM of HMME or AE in the dark for 30 min, and irradiated with 400–780 nm white light for 15 min (72 J cm^–2^). After treatment, fungal cells were centrifuged at 4,000 rpm for 10 min, resuspended in 2 mL of sterile PBS containing 1 μg/mL of Hoechst 33342 (Aladdin, China), and incubated at room temperature for 10 min in the dark. After three times wash with PBS, fungal samples were pipetted onto glass slide, immobilized by coverslip, and recorded on a fluorescence microscope (Zeiss Scope.A1, Germany). The fungal cells without light irradiation were used as control.

### Intracellular Reactive Oxygen Species Level

To evaluate the generation of intracellular ROS induced by HMME or AE-mediated aPDT, a ROS probe 2′,7′-dichlorohydrofluorescein diacetate (H_2_DCFDA, Aladdin, China) was utilized. Firstly, H_2_DCFDA stock solution was prepared by dissolving 1 mg of H_2_DCFDA in 400 μL DMSO. Next, fungal cells of clinical isolate 1 and isolate 2 (1 × 10^7^ CFU/mL) were collected by centrifugation and divided into two groups. The first group cells were incubated with 1 and 10 μM of HMME or AE in the dark for 30 min, respectively, and irradiated with 400–780 nm white light for 0.5 min (2.4 J cm^–2^). The second group cells were incubated with 10 μM of HMME or AE in the dark for 30 min and irradiated with 400–780 nm white light for 0.5 and 5 min (2.4 J cm^–2^ and 24 J cm^–2^), respectively. After treatments, 10 μL of H_2_DCFDA stock solution was added. Following incubation at 37°C in the dark for 30 min and wash with PBS for two times, intracellular ROS of *M. furfur* cells was analyzed by a flow cytometry (Beckman CytoFLEX, China). The positive control was defined as *M. furfur* cells treated with 10 mM of H_2_O_2_ for 1 h. Each experiment was performed independently for three times.

### Protease Assay

The protease activity of *M. furfur* cells after aPDT treatments was evaluated by the whole milk plate method (Coutinho and Paula, 2000). Fungal cells of clinical isolate 1 and isolate 2 (1 × 10^7^ CFU/mL) were incubated with 0, 1, 5, and 10 μM of HMME or AE in the dark for 30 min, respectively, and irradiated with 400–780 nm white light for 20 min (96 J cm^–2^). After treatments, 20 μL of fungal suspension was added into the holes of whole milk agar plates (containing 40 g/L glucose, 20 g/L agar, and 5% pasteurized whole milk), which were sealed with Parafilm and incubated at 37°C for another 7 days. After fixing with 10% trichloroacetic acid for 2 h, the whole milk plates were stained with Coomassie blue overnight and decolorized subsequently. The protease activity was evaluated by measuring the diameter of the transparent ring around the holes. Each experiment was performed independently for three times.

### Lipase Assay

The lipase assay was performed according to a previously reported study (Juntachai et al., 2009). In brief, fungal cells of clinical isolate 1 and isolate 2 (1 × 10^7^ CFU/mL) were incubated with 0, 1, 5, and 10 μM of HMME or AE in the dark for 30 min, respectively, and irradiated with 400–780 nm white light for 20 min (96 J cm^–2^). After treatments, *M. furfur* cells were transferred into olive oil medium (containing 40 g/L glucose, 10 g/L peptone, 0.05 g/L chloramphenicol, 0.1 g/L yeast extract, 0.2% Tween 80, 4% olive oil, and 2.5 g/L glycerin monostearate) and cultured at 37°C for 4 h. Next, the culture medium was centrifuged at 4,000 rpm for 5 min, and the supernatant was collected and filtered through a 0.22-μm membrane (Merck Millipore, United States). Next, 10 μL of the supernatant was added into 100 μL of citrate buffer (100 mM, pH = 5.0) containing 0.5 mM 4-nitrophenylpalmitate (4-NPP) and 0.5% TritonX-100. And 200 μL of Tris–HCl (1 M, pH = 8.0) was added into the above mixture to terminate the reaction. The lipase activity was measured spectro-photometrically at 405 nm based on releasing of 4-nitrophenol (4-NP) from 4-NPP. The concentration of released 4-NP was determined by a standard curve. Each experiment was performed independently for three times.

### Data Analysis

Three independent experiments were performed where described. The data were analyzed by graph pad prism software and shown as the mean ± standard error of the mean. Data were compared using one-way or two-way ANOVA, and the difference was considered significant in the case of *P* < 0.05.

## Results

### Photodynamic Antimicrobial Efficacy of Haematoporphyrin Monomethyl Ether and Aloe Emodin

**FIGURE 2:**
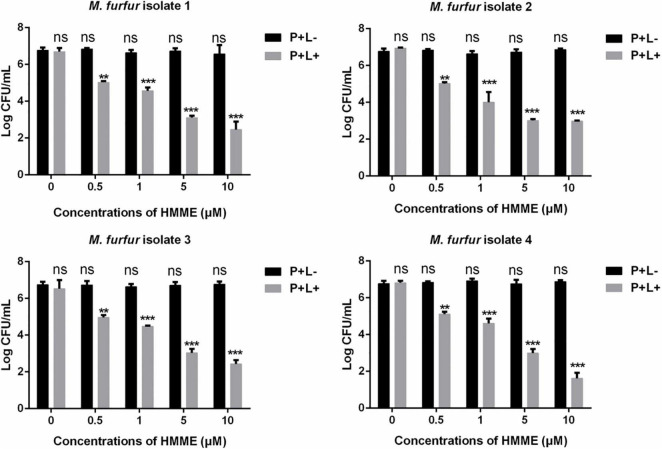
Survival of *M. furfur* clinical isolates after aPDT mediated by HMME. (P+L–) represents the *M. furfur* cells treated with different concentrations of HMME in the dark; (P+L+) represents the *M. furfur* cells treated with different concentrations of HMME and irradiated with 96 J cm^–2^ of light. Each value refers to mean ± standard deviation (SD; *n* = 3; ns: no significance, **P < 0.01, ***P < 0.001).

Fungal cells of the four clinical isolates were firstly incubated with 0, 0.5, 1, 5, and 10 μM of HMME in the dark for 30 min, respectively, and irradiated with 96 J cm^–2^ of light. As shown in Figure 2, irradiation with 96 J cm^–2^ of light had no significant cytotoxic effect on *M. furfur* cells of the four clinical isolates (0 μM HMME), and the fungal survival decreased with the increasing concentrations of HMME. For clinical isolate 1, 0.5, 1, 5, and 10 μM of HMME in the presence of 96 J cm^–2^ light irradiation yielded 1.90, 2.50, 3.79, and 4.31 log_10_ reductions in fungal survival, respectively. For clinical isolate 2, the same concentrations of HMME in the presence of 96 J cm^–2^ light irradiation yielded 1.73, 2.99, 3.98, and 4.12 log_10_ reductions in fungal survival, respectively. For clinical isolate 3, the same concentrations of HMME in the presence of 96 J cm^–2^ light irradiation yielded 1.80, 2.29, 3.73, and 4.34 log_10_ reductions in fungal survival, respectively. For clinical isolate 4, the same concentrations of HMME in the presence of 96 J cm^–2^ light irradiation yielded 1.65, 2.16, 3.77, and 5.15 log_10_ reductions in fungal survival, respectively.

**FIGURE 3:**
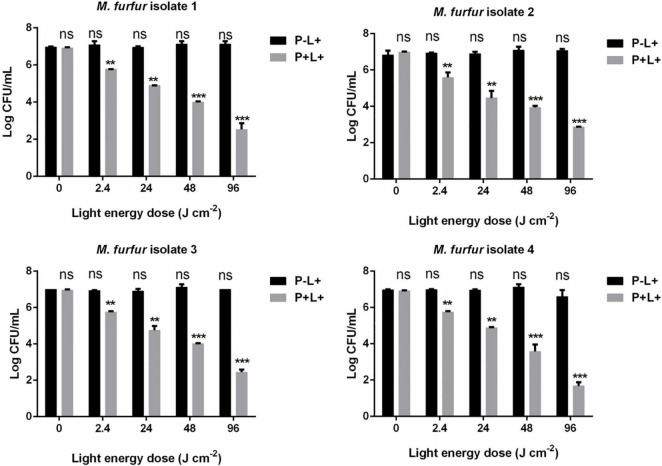
. Survival of *M. furfur* clinical isolates after aPDT mediated by HMME. (P–L+) represents the *M. furfur* cells irradiated with different energy doses of light; (P+L+) represents the *M. furfur* cells treated with 10 μM of HMME and irradiated with different energy doses of light. Each value refers to mean ± standard deviation (SD; *n* = 3; ns: no significance, **P < 0.01, ***P < 0.001).

To evaluate the effect of light energy doses on antimicrobial efficacy of HMME-mediated aPDT, *M. furfur* cells of the four clinical isolates were incubated with 10 μM HMME in the dark for 30 min and irradiated with 0, 2.4, 24, 48, and 96 J cm^–2^ of light. As shown in Figure 3, incubation with 10 μM HMME in the dark for 30 min also showed no dark toxicity on *M. furfur* cells of the four clinical isolates (0 J cm^–2^). With the increasing light energy doses, the fungal survival decreased correspondingly. For clinical isolate 1, 10 μM HMME followed by irradiation with 0, 2.4, 24, 48, and 96 J cm^–2^ of light decreased the fungal survival by 1.23, 2.20, 2.96, and 4.47 log_10_, respectively. For clinical isolate 2, 10 μM HMME followed by irradiation with the same light energy doses decreased the fungal survival by 1.82, 2.83, 3.10, and 3.99 log_10_, respectively. For clinical isolate 3, 10 μM HMME followed by irradiation with the same light energy doses decreased the fungal survival by 1.03, 2.24, 2.77, and 4.35 log_10_, respectively. For clinical isolate 4, 10 μM HMME followed by irradiation with the same light energy doses decreased the fungal survival by 1.03, 1.89, 3.57, and 5.16 log_10_, respectively.

**FIGURE 4:**
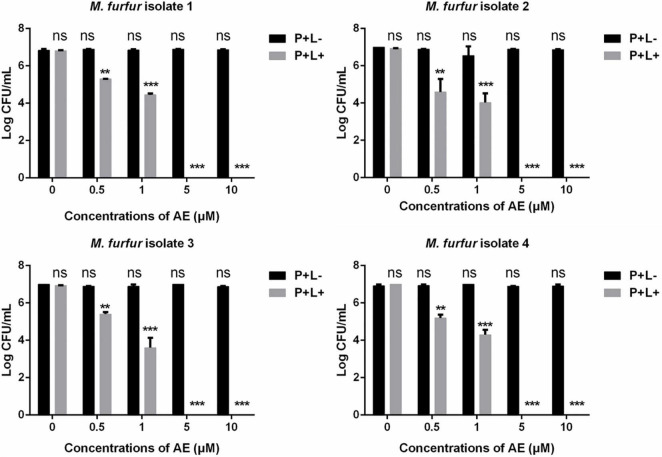
Survival of *M. furfur* clinical isolates after aPDT mediated by AE. (P+L–) represents the *M. furfur* cells treated with different concentrations of AE in the dark; (P+L+) represents the *M. furfur* cells treated with different concentrations of AE and irradiated with 96 J cm^–2^ of light. Each value refers to mean ± standard deviation (SD; *n* = 3; ns: no significance, **P < 0.01, ***P < 0.001).

Next, a second PS AE was employed. As shown in Figure 4, 0.5 and 1 μM of AE in the presence of 96 J cm^–2^ light irradiation achieved 1.71 and 2.60 log_10_ reductions in fungal survival for clinical isolate 1, respectively. For clinical isolate 2, the same concentrations of AE in the presence of 96 J cm^–2^ light irradiation achieved 2.23 and 3.02 log_10_ reductions in fungal survival, respectively. For clinical isolate 3, the same concentrations of AE in the presence of 96 J cm^–2^ light irradiation achieved 1.37 and 3.17 log_10_ reductions in fungal survival, respectively. For clinical isolate 4, the same concentrations of AE in the presence of 96 J cm^–2^ light irradiation achieved 1.57 and 2.47 log_10_ reductions in fungal survival, respectively. Under the same light energy dose (96 J cm^–2^), 5 and 10 μM of AE were able to kill all *M. furfur* cells of the four clinical isolates, achieving 7.00 log_10_ reduction in fungal survival.

**FIGURE 5:**
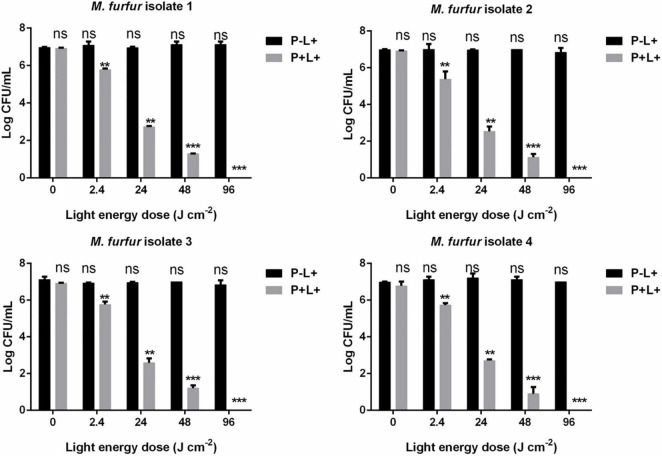
Survival of *M. furfur* clinical isolates after aPDT mediated by AE. (P–L + ) represents the *M. furfur* cells irradiated with different energy doses of light; (P + L + ) represents the *M. furfur* cells treated with 10 μM of AE and irradiated with different energy doses of light. Each value refers to mean ± standard deviation (SD; *n* = 3; ns: no significance, **P < 0.01, ***P < 0.001).

The effect of light energy doses on antimicrobial efficacy of AE-mediated aPDT was also investigated. As shown in Figure 5, no significant dark toxicity was induced by 10 μM AE (0 J cm^–2^). For clinical isolate 1, 2.4, 24, and 48 J cm^–2^ irradiation in the presence of 10 μM AE reduced the fungal survival by 1.23, 4.30, and 5.70 log_10_, respectively. For clinical isolate 2, the same light energy doses in the presence of 10 μM AE reduced the fungal survival by 2.06, 4.57, and 5.94 log_10_, respectively. For clinical isolate 3, the same light energy doses in the presence of 10 μM AE reduced the fungal survival by 1.14, 4.39, and 5.59 log_10_, respectively. For clinical isolate 4, the same light energy doses in the presence of 10 μM AE reduced the fungal survival by 1.26, 4.07, and 6.14 log_10_, respectively. When the light energy dose reached 96 J cm^–2^, no viable *M. furfur* cell of the four clinical isolates was observed.

### Fluorescence Imaging

**FIGURE 6:**
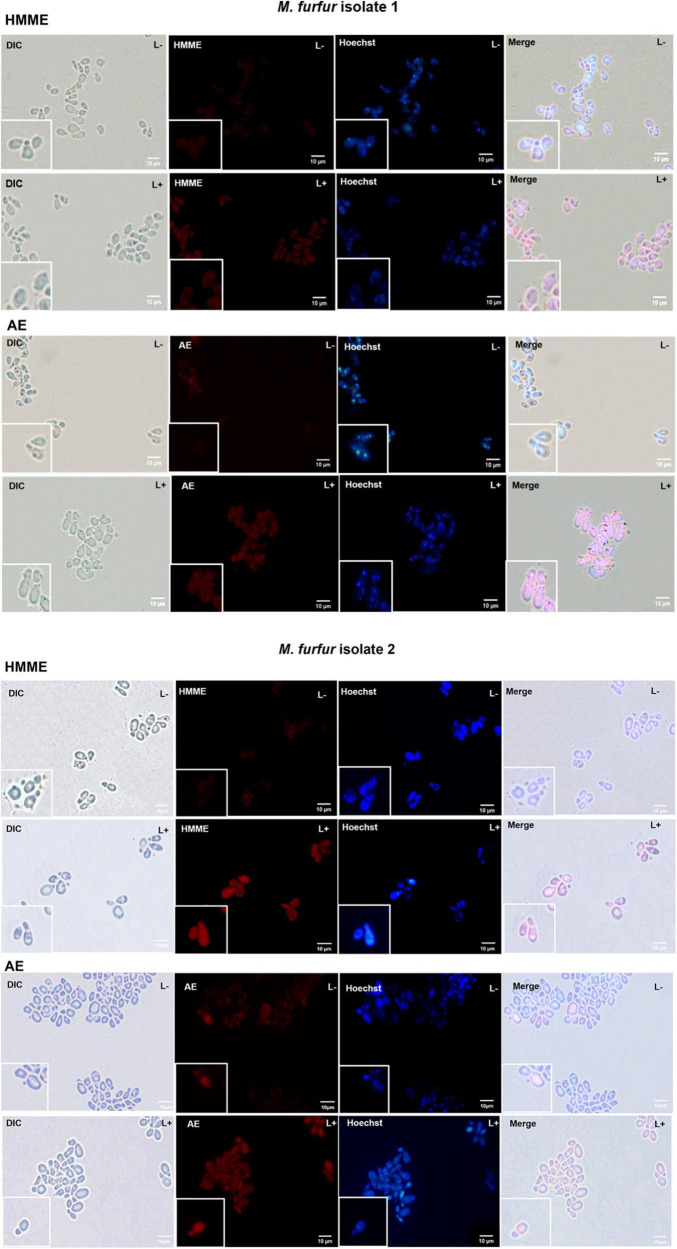
Fluorescence images of *M. furfur* cells after HMME and AE-mediated aPDT. (L–) represents the *M. furfur* cells treated with 10 μM of PSs; (L+) represents the *M. furfur* cells treated with 10 μM of PSs and irradiated with 72 J cm^–2^ of light.

To investigate the uptake of HMME and AE by *M. furfur*, fungal cells of the clinical isolate 1 and isolate 2 were treated with 10 μM of HMME or AE and stained with a DNA-specific fluorescent dye Hoechst 33342, and the fluorescence images were shown in Figure 6. Without light irradiation, a weak red fluorescence from HMME or AE could be observed in the cells of *M. furfur* (HMME and AE, L-). The nucleus of *M. furfur* could be differentiated as punctate blue fluorescence (Hoechst 33342, L-). But following irradiation, a stronger red fluorescence of HMME or AE was detected in the entire cell (HMME and AE, L+). There was no significant difference in the blue fluorescence pattern of nucleus before and after light irradiation (Hoechst 33342, L+).

### Intracellular Reactive Oxygen Species

**FIGURE 7:**
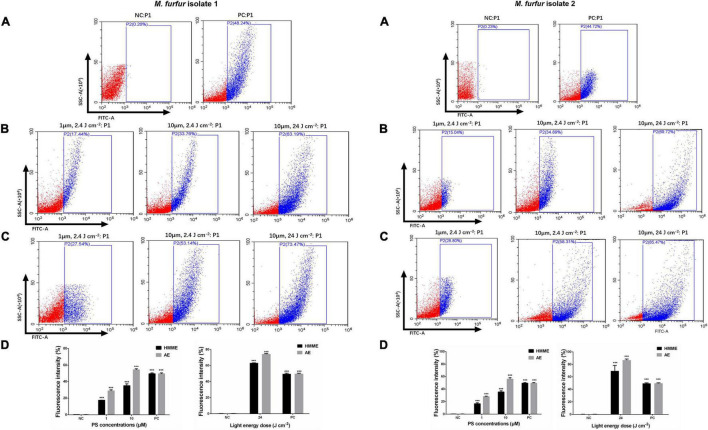
Intracellular ROS accumulation in *M. furfur* cells after HMME and AE-mediated aPDT. **(A)** Fluorescence intensities of DCF in *M. furfur* cells (NC: without any treatment, PC: treated with 10 mM H_2_O_2_ for 1 h); **(B)** fluorescence intensities of DCF in *M. furfur* cells treated with different concentrations of HMME and irradiated with different energy doses of light; **(C)** fluorescence intensities of DCF in *M. furfur* cells treated with different concentrations of AE and irradiated with different energy doses of light; **(D)** fluorescence intensities of DCF in *M. furfur* cells (NC: without any treatment, PC: cells treated with 10 mM H_2_O_2_ for 1 h). Each value refers to mean ± standard deviation (SD; *n* = 3; ns: no significance, ***P < 0.001).

The intracellular ROS level was shown in Figure 7. As the positive control, incubation with 10 mM H_2_O_2_ for 1 h increased the ROS level by 48.24 and 44.72% for *M. furfur* isolate 1 (Figure 7A) and isolate 2 (Figure 7B), respectively. For clinical isolate 1, 1 and 10 μM of HMME in the presence of 2.4 J cm^–2^ light irradiation increased the intracellular ROS level by 17.44 and 23.76%, respectively, and the same concentrations of AE induced an increase of intracellular ROS level by 27.54 and 53.14%, respectively (Figure 7A). For clinical isolate 2, 1 and 10 μM of HMME in the presence of 2.4 J cm^–2^ light irradiation increased the intracellular ROS level by 16.69 and 35.47%, respectively, and the same concentrations of AE induced an increase of intracellular ROS level by 28.05 and 55.93%, respectively (Figure 7B). When irradiated with 24 J cm^–2^ light, 10 μM HMME and AE enhanced the intracellular ROS level by 63.19 and 73.47% for clinical isolate 1, respectively (Figure 7A). Under the same light energy dose (24 J cm^–2^), 10 μM HMME and AE enhanced the intracellular ROS level by 69.24 and 86.49% for clinical isolate 2, respectively (Figure 7B).

### Protease Activity

**FIGURE 8:**
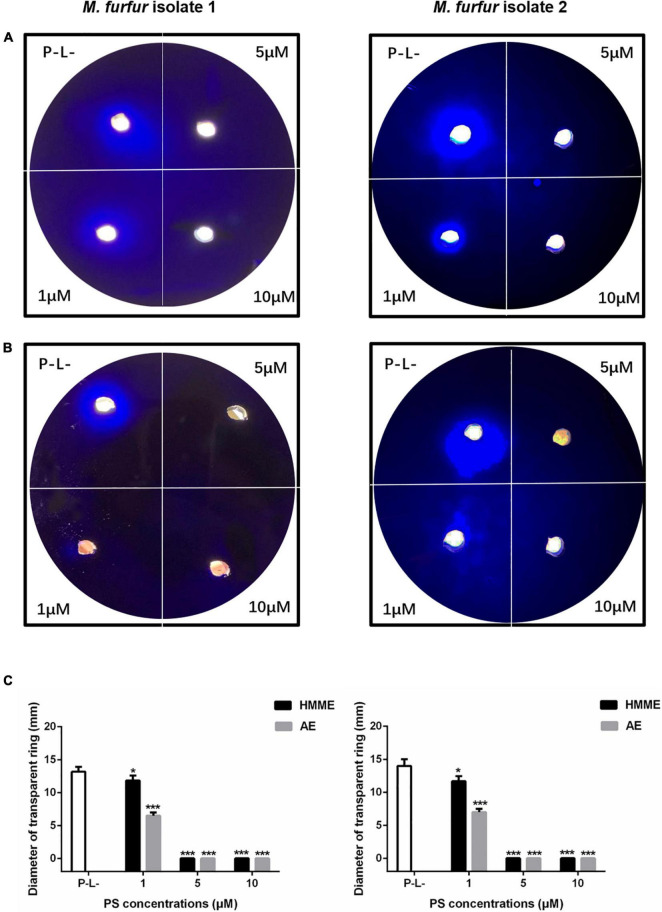
Secreted protease activity of *M. furfur* after HMME and AE-mediated aPDT. **(A)** *M. furfur* cells treated with different concentrations of HMME and irradiated with 96 J cm^–2^ of light and incubated in whole milk plate for 7 days (P-L-: without any treatment); **(B)** *M. furfur* cells treated with different concentrations of AE and irradiated with 96 J cm^–2^ of light and incubated in whole milk plate for 7 days (P-L-: without any treatment); and **(C)** diameters of transparent ring of *M. furfur* cultured in whole milk plate (P-L-: without any treatment). Each value refers to mean ± standard deviation (SD; *n* = 3; ns: no significance, *P < 0.05, ***P < 0.001).

To investigate the effect of HMME or AE-mediated aPDT on secreted protease activity of *M. furfur*, fungal cells of the clinical isolate 1 and isolate 2 were treated with 0, 1, 5, and 10 μM HMME or AE, irradiated with 96 J cm^–2^ light, and incubated in the holes of whole milk plates for 7 days. As shown in Figure 8, the transparent zone diameters of *M. furfur* cells without any treatment were around 14 mm. For clinical isolate 1, the diameters of transparent zone decreased by 16.0 and 50.4% in *M. furfur* cells treated with 1 μM HMME and AE in the presence of 96 J cm^–2^ light, respectively. For clinical isolate 2, the diameters of transparent zone decreased by 16.7 and 50.0% in *M. furfur* cells treated with 1 μM HMME and AE in the presence of 96 J cm^–2^ light, respectively.

When 5 and 10 μM HMME or AE were applied, the transparent zone was not observed, suggesting that the secreted protease activity of *M. furfur* was completely inhibited by HMME and AE-mediated aPDT.

### Lipase Activity

**FIGURE 9:**
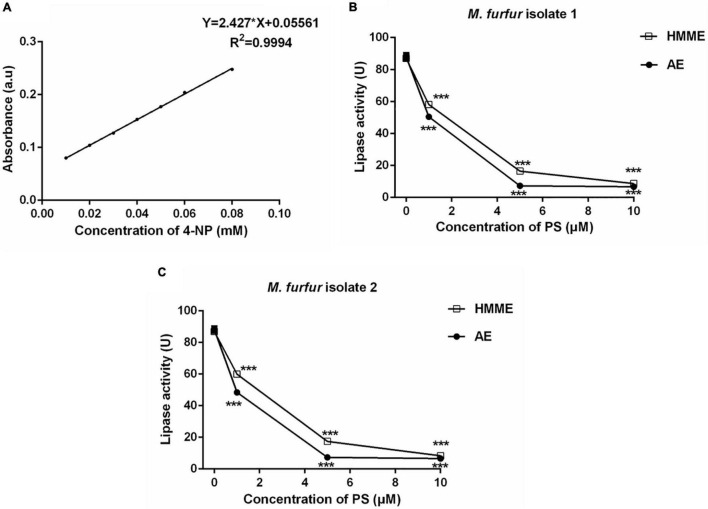
Secreted lipase activity of *M. furfur* after HMME and AE-mediated aPDT. **(A)** Standard curve of 4-NP; **(B)** lipase activity of *M. furfur* isolate 1 treated with different concentrations of PSs and irradiated with 96 J cm^–2^ of light; and **(C)** lipase activity of *M. furfur* isolate 2 treated with different concentrations of PSs and irradiated with 96 J cm^–2^ of light. One unit of lipase activity is defined as the amount of 4-NP nmol h^–1^ released from 4-NPP at 30°C. Each value refers to mean ± standard deviation (SD; *n* = 3; ns: no significance, ***P < 0.001).

To investigate the effect of HMME or AE-mediated aPDT on secreted lipase activity of *M. furfur*, fungal cells of the clinical isolate 1 and isolate 2 were treated with 0, 1, 5, and 10 μM HMME or AE, irradiated with 96 J cm^–2^ light, and cultured in olive oil medium for another 4 h. The secreted lipase in the culture medium was collected and its activity was measured spectro-photometrically. The standard curve of 4-NP was provided in Figure 9A. For clinical isolate 1, 1, 5, and 10 μM HMME in the presence of 96 J cm^–2^ light decreased the secreted lipase activity of *M. furfur* by 33.15, 81.11, and 90.36%, respectively. Under irradiation with the same light energy dose, 1, 5, and 10 μM AE decreased the secreted lipase activity of *M. furfur* by 42.69, 91.71, and 92.37%, respectively (Figure 9B). For clinical isolate 2, 1, 5, and 10 μM HMME in the presence of 96 J cm^–2^ light decreased the secreted lipase activity of *M. furfur* by 31.05, 79.99, and 90.37%, respectively (Figure 9C). Under irradiation with the same light energy dose, 1, 5, and 10 μM AE decreased the secreted lipase activity of *M. furfur* by 44.33, 91.51, and 92.43%, respectively (Figure 9C).

## Discussion

Photodynamic therapy (PDT) has been already used as therapeutic regimen for oncologic skin diseases such as actinic keratosis, basal cell carcinoma, squamous cell carcinoma *in situ*, and Bowen’s disease (Kim et al., 2015). A great number of studies also indicated that PDT or aPDT was effective for the treatment of acne vulgaris, psoriasis, rosacea, viral warts, tinea pedis, tinea cruris, and onychomycosis in dermatology (Kim et al., 2015; Baltazar et al., 2016). In infectious skin diseases, *M. furfur* can cause *Malassezia* folliculitis, pityriasis versicolor, seborrheic dermatitis, and some forms of atopic dermatitis under specific conditions (Saunte et al., 2020). But the efficacy of aPDT treatment against these *M. furfur*-related skin diseases has been rarely reported.

Lee et al. (2010, 2011) previously employed methyl aminolevulinate (MAL)-mediated aPDT to treat patients with recalcitrant *Malassezia* folliculitis, and found that inflammatory lesions obviously decreased and improved after three sessions of MAL-mediated aPDT. In another study, Kin and Kim (2007) indicated that *M. furfur*-caused pityriasis versicolor was completely cured after two sessions of 5-aminolevulinic acid (ALA)-mediated aPDT. Kwon et al. (2014) treated 23 patients with *M. furfur*-induced facial seborrhoeic dermatitis using indole-3-acetic acid (IAA)-mediated aPDT. After three sessions of photodynamic treatments, Seborrhoeic dermatitis Area and Severity Index (SASI) and total symptom were significantly improved, and sebum excretion was significantly reduced. Painless and no adverse effect were observed during the treatment process (Kwon et al., 2014). These clinical trials demonstrated that aPDT is a promising and effective alternative for the treatment of *M. furfur*-caused skin diseases.

PS is a key factor in the photodynamic treatment process, and the porphyrin-based PSs are known as the earliest and most useful PSs in clinical trials (Jia et al., 2017a). Among them, protoporphyrin IX (PpIX) has been widely used in photodynamic research or clinical trials since it is very safe and is the key component of hemoglobin in red blood cells (Jia et al., 2017a). PpIX has the singlet oxygen quantum yield of 0.56 and maximum absorption of 410 nm, along with four smaller peaks near 510, 540, 580, and 635 nm (Redmond and Gamlin, 1999; O’Connor et al., 2009). HMME is a porphyrin-based PS developed in China by the early 1990s, which consists of two positional isomers: 3-(1-methyloxyethyl)-8-(1-hydroxyethyl) deuteroporphyrin IX and 8-(1-methyloxyethyl)-3-(1-hydroxyethyl) deuteroporphyrin IX. In comparison with the commonly used PpIX, HMME exhibits similar chemical structure, absorption peaks (400, 503, 539, 565, and 617 nm), and singlet oxygen quantum yield (0.6; Lei et al., 2012). Although HMME has been approved in China for the photodynamic treatment of naevus flammeus, showing high effectiveness and safety (Mei et al., 2019), it is still not expanded in the photodynamic treatment of bacteria or fungi induced skin diseases. AE is a newly discovered natural anthraquinone PS isolated from Chinese traditional herbs, possessing absorption in the blue region (430 nm) and singlet oxygen quantum yield of 0.57, which may be suitable for the treatment of superficial lesions (Zang et al., 2017). It was reported that AE had low cytotoxicity on epidermis and hypodermal cells and it caused no obvious effects on the weight and pathological changes of mice in our previous study (Wang et al., 2021). Thus, both HMME and AE could serve as potential PSs against *M. furfur.*

The present work firstly investigated the photodynamic antimicrobial efficacy of the two PSs from China against clinical isolates of *M. furfur*. The obtained results showed that both HMME and AE had no significant dark toxicity on *M. furfur* cells, but in the presence of light irradiation they effectively inactivated *M. furfur* cells in a PS concentration and light energy dose-dependent manner. Additionally, AE exhibited higher photodynamic antimicrobial efficacy against *M. furfur* than HMME. In the presence of 96 J cm^–2^ light irradiation, while 10 μM HMME induced a reduction of about 5 log_10_ in survival of *M. furfur*, 5 μM AE was able to kill all *M. furfur* cells (7 log_10_ reduction). We speculated that the difference had relationship with the polarities of these two PSs. AE is a hydrophobic PS and *M. furfur* is a lipid-dependent yeast that might uptake more hydrophobic PS. But HMME is more soluble in aqueous solution compared to AE. To support this speculation, more investigations are needed in our future studies.

The oxidative damage induced by aPDT mainly occurred on the cellular plasma membrane and/or nucleic acids (Plotino et al., 2019). The fluorescence microscopy revealed that stronger fluorescence of HMME and AE could be detected throughout the entire cytoplasm of *M. furfur* cells after light irradiation, suggesting that the cell envelop of fungal cells might be disturbed during the aPDT process. We also found that the blue fluorescence pattern of Hoechst 33342 showed no significant difference in the intracellular localization before and after light irradiation, indicating that HMME and AE-mediated aPDT had inapparent effect on *M. furfur* nucleus.

Intracellular ROS production can cause severe oxidative stress within cells through the formation of oxidized cellular macromolecules (Rajendran, 2016). We used H_2_DCFDA as a ROS probe to evaluate the level of intracellular ROS in *M. furfur* cells induced by aPDT. The results demonstrated that the intracellular ROS significantly increased after both HMME and AE-mediated aPDT. Additionally, our data showed that the intracellular ROS level induced by AE-mediated aPDT was higher than that induced by HMME-mediated aPDT, which was in accordance with the antimicrobial efficacy of aPDT mediated by the two PSs.

Secretory hydrolytic enzymes such as protease and lipase of *M. furfur* play an important role in fungal growth and maintaining the function of fungal cell membrane (Mancianti et al., 2000). These enzymes have also been assumed as virulence factors because they are related to invasive fungal infections or development of inflammation (Juntachai and Kajiwara, 2015). It has been reported that protease could alter the permeability of the epithelial barrier and induce inflammatory responses, and lipase could favur the invasion of host tissue (Mancianti et al., 2000; Park et al., 2013). The present study for the first time found that aPDT could effectively inhibit the activity of extracellular protease and lipase of *M. furfur*. After treatment with 5 μM HMME or AE and irradiation with 96 J cm^–2^ light, the secreted protease activity of *M. furfur* was completely inhibited. And 10 μM HMME or AE in the presence of 96 J cm^–2^ light irradiation decreased lipase activity of *M. furfur* by over 90%.

One limitation of this study is that we only investigated the photodynamic antimicrobial efficacy of HMME and AE on *M. furfur in vitro*, but the treatment efficacy of aPDT mediated by the two PSs for *M. furfur*-caused dermatological diseases still remains unclear. Nalamothu et al. (2009) previously established a *M. furfur*-infected skin model of guinea pig by removing the dorsal hair of animal and inoculating *M. furfur* suspension to the intact dorsal skin once a day for seven consecutive days. Granger et al. (2019) recently developed a novel *ex vivo* model of seborrheic dermatitis based on human skin explants inoculated with *M. furfur*. We will utilize these models to evaluate the antimicrobial efficacy of HMME and AE-mediated aPDT against *M. furfur in vivo* and *ex vivo* in our ongoing studies.

## Conclusion

This study investigated the photodynamic effect of aPDT mediated by two PSs from China on clinical isolates of *M. furfur in vitro* and expanded the knowledge regarding to photodynamic inactivation of fungi. Both HMME and AE exhibited no significant dark toxicity on *M. furfur*; but in the presence of light, they effectively inactivated the fungal cells in a PS concentration and light energy dose-dependent manner. Under the same irradiation condition, AE showed higher photodynamic antimicrobial efficacy against *M. furfur* than HMME. Fluorescence imaging and ROS measurement revealed that HMME and AE-mediated aPDT disturbed the fungal cell envelop and significantly increased the intracellular ROS level. Besides, aPDT mediated by HMME and AE could effectively inhibit the activity of secreted protease and lipase of *M. furfur* cells. Although these obtained results demonstrated that HMME and AE could serve as promising PSs for the photodynamic treatment of dermatological diseases caused by *M. furfur*, further *in vivo* and *ex vivo* investigations are needed in our future studies to determine that HMME and AE can meet the clinical practice requirements.

## Data Availability Statement

The original contributions presented in the study are included in the article/supplementary material, further inquiries can be directed to the corresponding author/s.

## Author Contributions

CL, FY, and SG conceived and designed the study. ZC and MZ conducted most of the experiments and prepared the figures. XN and YZ collected and characterized the clinical isolate of *M. furfur*. XW analyzed the data. CL and FY drafted and revised the manuscript. SG provided general guidance. All authors read and approved the final manuscript.

## Conflict of Interest

The authors declare that the research was conducted in the absence of any commercial or financial relationships that could be construed as a potential conflict of interest.

## Publisher’s Note

All claims expressed in this article are solely those of the authors and do not necessarily represent those of their affiliated organizations, or those of the publisher, the editors and the reviewers. Any product that may be evaluated in this article, or claim that may be made by its manufacturer, is not guaranteed or endorsed by the publisher.

## References

[B1] Ashbee H. R. Evans E. G. (2002). Immunology of diseases associated with *Malassezia* species. *Clin. Microbiol. Rev.* 15 21–57. 10.1128/CMR.15.1.21-57.2002 11781265 PMC118058

[B2] Baltazar L. M. Ray A. Santos D. A. Cisalpino P. S. Friedman A. J. Nosanchuk J. D. (2016). Antimicrobial photodynamic therapy: an effective alternative approach to control fungal infections. *Front. Microbiol.* 6:202. 10.3389/fmicb.2015.00202 25821448 PMC4358220

[B3] Cafarchia C. Gasser R. B. Figueredi L. A. Latrofa M. S. Otranto D. (2011). Advances in the identification of *Malassezia*. *Mol. Cell Probes*. 25 1–7. 10.1016/j.mcp.2010.12.003 21193026

[B4] Cafarchia C. Iatta R. Immediatio D. Puttilli M. R. Otranto D. (2015). Azole susceptibility of *Malassezia pachydermatis* and *Malassezia furfur* and tentative epidemiological cut-off values. *Med. Mycol.* 53 743–748. 10.1093/mmy/myv049 26162472

[B5] Calzavara-Pinton P. Rossi M. T. Sala R. Venturini M. (2012). Photodynamic antifungal chemotherapy. *Photochem. Photobiol*. 88 512–522. 10.1111/j.1751-1097.2012.01107.x 22313493

[B6] Chen D. Xu H. (2012). Isolation and identification of *Malassezia furfur* from lower respiratory tract secretions. *Chin. J. Lab. Med*. 35 711–715.

[B7] Coutinho S. D. Paula C. R. (2000). Proteinase, phospholipase, hyaluronidase and chondroitin-sulphatase production by *Malassezia* pachydermatis. *Med. Mycol.* 38 73–76. 10.1080/mmy.38.1.73.76 10746230

[B8] Dai T. Fuchs B. B. Coleman J. J. Parate R. A. Astrakas C. Denis T. G. (2012). Concepts and principles of photodynamic therapy as an alternative antifungal discovery platform. *Front. Microbiol*. 3:120. 10.3389/fmicb.2012.00120 22514547 PMC3322354

[B9] Dong X. Zeng Y. Liu Y. You L. Yin X. Fu J. (2020). Aloe-emodin: A review of its pharmacology, toxicity, and pharmacokinetics. *Rhytother. Res.* 34 270–281. 10.1002/ptr.6532 31680350

[B10] Gaitanis G. Magiatis P. Hantschke M. Bassukas I. D. Velegraki A. (2012). The *Malassezia* genus in skin and systemic diseases. *Clin. Microbiol. Rev.* 25 106–141. 10.1128/CMR.00021-11 22232373 PMC3255962

[B11] Gao Z. Perez-Perez G. I. Chen Y. Blaser M. J. (2010). Quantitation of major human cutaneous bacterial and fungal populations. *J. Clin. Microbiol.* 48 3575–3581. 10.1128/JCM.00597-10 20702672 PMC2953113

[B12] Granger C. Balato A. Goñi-de-Cerio F. Garre A. Narda M. (2019). Novel non-steroidal facial cream demonstrates antifungal and anti-inflammatory properties in *ex vivo* model for seborrheic dermatitis. *Dermatol. Ther.* 9 571–578. 10.1007/s13555-019-0311-4 31278482 PMC6704224

[B13] Hamblin M. R. Hasan T. (2004). Photodynamic therapy: a new antimicrobial approach to infectious disease? *Photochem. Photobiol. Sci*. 3 436–450. 10.1039/b311900a 15122361 PMC3071049

[B14] Iatta R. Puttilli M. R. Immediatio D. Otranto D. Cafarchia C. (2017). The role of drug efflux pumps in *Malassezia pachydermatis* and *Malassezia furfur* defence against azoles. *Mycoses*. 60 178–182. 10.1111/myc.12577 27774659

[B15] Jia H. R. Jiang Y. W. Zhu Y. X. Li Y. H. Wang H. Y. Han X. F. (2017a). Plasma membrane activatable polymeric nanotheranostics with self-enhanced light-triggered photosensitizer cellular influx for photodynamic cancer therapy. *J. Control. Release* 255 231–241. 10.1016/j.jconrel.2017.04.030 28442408

[B16] Jia H. R. Zhu Y. X. Chen Z. Wu F. G. (2017b). Cholesterol-assisted bacterial cell surface engineering for photodynamic inactivation of gram-positive and gram-negative bacteria. *ACS Appl. Mater. Interfaces.* 9 15943–15951. 10.1021/acsami.7b02562 28426936

[B17] Juntachai W. Kajiwara S. (2015). Differential expression of extracellular lipase and protease activities of mycelial and yeast forms in *Malassezia furfur*. *Mycopathologia* 180 143–151. 10.1007/s11046-015-9900-7 26173769

[B18] Juntachai W. Oura T. Murayama S. Y. Kajiwara S. (2009). The lipolytic enzymes activities of *Malassezia* species. *Med. Mycol.* 47 477–484. 10.1080/13693780802314825 18798119

[B19] Kashef N. Hamblin M. R. (2017). Can microbial cells develop resistance to oxidative stress in antimicrobial photodynamic inactivation? *Drug Resist. Updat.* 31 31–42. 10.1016/j.drup.2017.07.003 28867242 PMC5673603

[B20] Kim M. Jung H. Y. Park H. J. (2015). Topical PDT in the treatment of benign skin diseases: Principles and new applications. *Int. J. Mol. Sci*. 16 23259–23278. 10.3390/ijms161023259 26404243 PMC4632697

[B21] Kin Y. J. Kim Y. C. (2007). Successful treatment of pityriasis versicolor with 5-aminolevulinic acid photodynamic therapy. *Arch. Dermatol*. 143 1218–1219. 10.1001/archderm.143.9.1218 17875898

[B22] Kwon S. H. Jeong M. Y. Park K. C. Youn S. W. Huh C. H. Na J. I. A. (2014). new therapeutic option for facial seborrheic dermatitis: indole-3-acetic acid photodynamic therapy. *J. Euro. Acad. Dermatol. Venereol.* 28 94–99. 10.1111/jdv.12070 23302041

[B23] Lee J. W. Kin B. J. Kim M. N. (2010). Photodynamic therapy: new treatment for recalcitrant *Malassezia* Folliculitis. *Lases Surg. Med*. 42 192–196. 10.1002/lsm.20857 20166153

[B24] Lee J. W. Lee H. I. Kim M. N. Kin B. J. Chun Y. J. Kim D. (2011). Topical photodynamic therapy with methyl aminolevulinate may be an alternative therapeutic option for the recalcitrant *Malassezia* folliculitis. *Int. J. Dermatol.* 50 485–496. 10.1111/j.1365-4632.2009.04377.x 21413966

[B25] Lei T. C. Glazner G. F. Duffy M. Scherrer L. Pendyala S. Li B. (2012). Optical properties of hematoporphyrin monomethyl ether (HMME), a PDT photosensitizer. *Photodiagnosis Photodyn. Ther.* 9 232–242. 10.1016/j.pdpdt.2012.01.003 22959803

[B26] Li J. Sun W. Yang Z. Gao G. Ran H. H. Xu K. F. (2020). Rational design of self-assembled cationic porphyrin-based nanoparticles for efficient photodynamic inactivation of bacteria. *ACS Appl. Mater. Interfaces*. 12 54378–54386. 10.1021/acsami.0c15244 33226224

[B27] Liu C. Hu M. Zeng X. Nair S. P. Xu J. (2016). Photodynamic inactivation of *Candida albicans* by hematoporphyrin monomethyl ether. *Future Microbiol.* 11 351–362. 10.2217/fmb.15.142 26933758

[B28] Ma W. Liu C. Li J. Hao M. Ji Y. Zeng X. (2020). The effects of aloe emodin-mediated antimicrobial photodynamic therapy on drug-sensitive and resistant *Candida albicans*. *Photochem. Photobiol. Sci.* 19 485–494. 10.1039/C9PP00352E 32232258

[B29] Ma W. Zhang M. Cui Z. Wang X. Niu X. Zhu Y. (2021). Aloe-emodin-mediated antimicrobial photodynamic therapy against dermatophytosis caused by *Trichophyton rubrum*. *Microb. Biotechnol.* 2021:13875. 10.1111/1751-7915.13875 34165875 PMC8867962

[B30] Mancianti F. Rum A. Nardoni S. Corazza M. (2000). Extracellular enzymatic activity of *Malassezia* spp. isolates. *Mycopathologia* 149 131–135. 10.1023/A:1007237408748 11307595

[B31] Mei Y. Xiao X. Fan L. Liu Q. Zheng M. Hamblin M. R. (2019). *In vitro* photodynamic therapy of endothelial cells using hematoporphyrin monomethyl ether (Hemoporfin): relevance to treatment of port wine stains. *Photodiagnosis Photodyn. Ther.* 27 268–275. 10.1016/j.pdpdt.2019.06.003 31185325 PMC6708484

[B32] Nalamothu V. O’Leary A. L. Kandavilli S. Fraser J. Pandya V. (2009). Evaluation of a nonsteroidal topical cream in a guinea pig model of *Malassezia furfur* infection. *Clin. Dermatol*. 27 S41–S43. 10.1016/j.clindermatol.2009.09.003 19878779

[B33] O’Connor A. E. Gallagher W. M. Byrne A. T. (2009). Porphyrin and nonporphyrin photosensitizers in oncology: preclinicaland clinical advances in photodynamic therapy. *Photochem. Photobiol*. 85 1053–1074. 10.1111/j.1751-1097.2009.00585.x 19682322

[B34] Pan T. Liu X. Liu C. Li J. Ma W. Qin Y. (2020). Evaluation of the photodynamic efficacy and effects of haematoporphyrin monomethyl ether on *Trichophyton rubrum* microconidia *in vitro*. *Mycoses* 63 1215–1225. 10.1111/myc.13149 32783251

[B35] Park M. Do E. Jung W. H. (2013). Lipolytic enzymes involved in the virulence of human pathogenic fungi. *Mycobiol*. 41 67–72. 10.5941/MYCO.2013.41.2.67 23874127 PMC3714442

[B36] Plotino G. Grande N. M. Mercade M. (2019). Photodynamic therapy in endodontics. *Int. Endod. J.* 52 760–774. 10.1111/iej.13057 30548497

[B37] Prohic A. Jovovic S. T. Krupalija-Fazlic M. Kuskunovic-Vlahovljak S. (2016). *Malassezia* species in healthy skin and in dermatological conditions. *Int. J. Dermatol*. 55 494–504. 10.1111/ijd.13116 26710919

[B38] Rajendran M. (2016). Quinones as photosensitizer for photodynamic therapy: ROS generation, mechanism and detection methods. *Photodiagnosis. Photodyn. Ther*. 13 175–187. 10.1016/j.pdpdt.2015.07.177 26241780

[B39] Redmond R. W. Gamlin J. N. A. (1999). Compilation of singlet oxygen yields from biologically relevant molecules. *Photochem. Photobiol*. 70 391–475. 10.1111/j.1751-1097.1999.tb08240.x 10546544

[B40] Rhimi W. Theelen B. Boekhout T. Aneke C. I. Otranto D. Cafarchia C. (2021). Conventional therapy and new antifungal drugs against *Malassezia* infections. *Med. Mycol*. 59 215–234. 10.1093/mmy/myaa087 33099634

[B41] Saunte D. M. L. Gaitanis G. Hay R. J. (2020). *Malassezia*-associated skin diseases, the use of diagnostics and treatment. *Front. Cell Infect. Microbiol.* 10:112. 10.3389/fcimb.2020.00112 32266163 PMC7098993

[B42] Spatz M. Richard M. L. (2020). Overview of the potential role of *Malassezia* in gut health and disease. *Front. Cell Infect. Microbiol.* 10:201. 10.3389/fcimb.2020.00201 32528901 PMC7265801

[B43] Takahashi H. Nakajima S. Sakata I. Iizuka H. (2014). Antifungal effect of TONS504-photodynamic therapy on *Malassezia furfur*. *J. Dermatol.* 41 895–897. 10.1111/1346-8138.12615 25226792

[B44] Tang Y. Xie H. Li J. Jian D. (2017). The association between treatment reactions and treatment efficiency of hemoporfin-photodynamic therapy on port wine stains: A prospective double blind randomized controlled trial. *Photodiagnosis. Photodyn. Ther.* 18 171–178. 10.1016/j.pdpdt.2017.02.005 28216012

[B45] Torres M. Cock de H. Ramírez A. M. C. (2020). *In vitro* or *in vivo* models, the next frontier for unraveling interactions between *Malassezia* spp. and hosts: How much do we know? *J. Fungi*. 6:155. 10.3390/jof6030155 32872112 PMC7558575

[B46] Wang Y. Li J. Geng S. Wang X. Cui Z. Ma W. (2021). Aloe-emodin-mediated antimicrobial photodynamic therapy against multidrug-resistant *Acinetobacter baumannii*: An *in vivo* study. *Photodiagnosis Photodyn. Ther.* 34:102311. 10.1016/j.pdpdt.2021.102311 33930578

[B47] Zang L. Zhao H. Ji X. Cao W. Zhang Z. Meng P. (2017). Photophysical properties, singlet oxygen generation efficiency and cytotoxic effects of aloe emodin as a blue light photosensitizer for photodynamic therapy in dermatological treatment. *Photochem. Photobiol. Sci*. 16 1088–1094. 10.1039/C6PP00453A 28530733

